# Efficacy of Sertraline Plus Placebo or Add-On Celecoxib in Major Depressive Disorder: Macrophage Migration Inhibitory Factor as a Promising Biomarker for Remission After Sertraline—Results From a Randomized Controlled Clinical Trial

**DOI:** 10.3389/fpsyt.2021.615261

**Published:** 2021-09-27

**Authors:** Maria S. Simon, Bianka Burger, Elif Weidinger, Gara Arteaga-Henríquez, Peter Zill, Richard Musil, Hemmo A. Drexhage, Norbert Müller

**Affiliations:** ^1^Department of Psychiatry and Psychotherapy, University Hospital, Ludwig-Maximilians-University, Munich, Germany; ^2^Marion von Tessin Memory-Center, Munich, Germany; ^3^Department of Psychiatry, Hospital Universitari Vall d'Hebron, Vall d'Hebron Research Institute (VHIR), Vall d'Hebron Barcelona Hospital Campus, Barcelona, Spain; ^4^Biomedical Network Research Centre on Mental Health (CIBERSAM), Madrid, Spain; ^5^Department of Immunology, Erasmus Medical Center, Rotterdam, Netherlands

**Keywords:** inflammatory, Major depressive disorder, cytokine, response, biomarker, anti-inflammatory treatment

## Abstract

**Introduction:** Previous research delivers strong indications that inflammatory activation leads to treatment resistance in a subgroup of patients with Major Depressive Disorder (MDD). Thus, tailored interventions are needed. The present study aimed to find potential biomarkers that may enable patients to be stratified according to immune activation.

**Methods:** A phase IIa randomized placebo-controlled trial was performed to assess levels of inflammatory compounds in responders/remitters and non-responders/non-remitters to sertraline plus celecoxib (*n* = 20) and sertraline plus placebo (*n* = 23). Levels of macrophage migration inhibitory factor, neopterin, and tumor necrosis factor alpha were determined by enzyme-linked immunosorbent assay; response and remission were measured by reduction of the Montgomery Åsberg Depression Rating Scale score.

**Results:** Both treatment groups showed a significant decline in depression symptoms, but no difference was found between groups. A clear pattern emerged only for macrophage migration inhibitory factor: placebo remitters showed significantly lower baseline levels than non-remitters (a similar trend was seen in responders and non-responders) while celecoxib responders showed a trend for higher baseline levels than non-responders.

**Conclusion:** Small subsample sizes are a notable limitation, wherefore results are preliminary. However, the present study provides novel insights by suggesting macrophage migration inhibitory factor as a promising biomarker for treatment choice.

The trial was registered in EU Clinical Trials Register (EU-CTR): https://www.clinicaltrialsregister.eu/ctr-search/trial/2009-011990-34/DE, EudraCT-No.: 2009-011990-34.

## Introduction

In clinical practice, patients' response to antidepressant treatment often remains unsatisfactory. Around 20% up to 50% of depressed patients show non-response to at least two standard antidepressant drug trials (1, 2). Furthermore, remission rates across different antidepressant treatment options are at 28% after initial treatment attempt and remission rates further decrease with each treatment failure (3). Thus, identifying patients prone to treatment resistance is important to enable early use of alternative treatment options. Previous research has consistently shown that inflammatory activation plays a role in the pathophysiology of Major Depressive Disorder (MDD) and pro-inflammatory activation has been implicated in treatment resistance to standard antidepressant medication in several studies (4). Results indicate that low-grade inflammation is present in a subgroup of MDD patients (5, 6) characterized by higher levels of circulating pro-inflammatory compounds (7–9). Overall, higher levels of these compounds were associated with depression, though results of single study show some variety (10–13). In particular, compounds such as C-reactive protein (CRP), Interleukin-6 (IL-6), and tumor necrosis factor alpha (TNFα) have been frequently described. Deficits in the T cell system and pro-inflammatory monocyte activation were also shown to be present in MDD patients (14–17).

Most studies investigate inflammatory markers from the periphery, while depressive symptoms result from dysregulations in the brain. Peripheral cytokines activate afferent nerves to the brain and can enter the brain themselves leading to further pro-inflammatory cytokine output by microglia in the brain (18–20). Besides the above-described parameters, markers of monocyte/macrophage activation (and endothelial function) have also been associated with MDD: Circulating levels of macrophage migration inhibitory factor (MIF) and neopterin were increased in depression as compared to healthy controls (21, 22). Monocytes are an important compound of the innate immune system because they are drivers of inflammation by releasing pro-inflammatory cytokines (23). Interestingly, multiple studies have confirmed a comorbidity between MDD and cardiovascular disease (24–26), and the link between MDD, low-grade inflammation, and cardiovascular events is highly suggested pointing to shared biological underpinnings (27). Thus, the use of anti-inflammatory drugs in MDD seems to be a reasonable approach to increase responsiveness. In patients with atherosclerosis, cyclooxygenase-(COX-)2, prostaglandin E receptors, and prostaglandin E synthase-1 were overexpressed in plaques and peripheral blood mononuclear cells [PBMCs; (28)]. Furthermore, stimulated macrophages exposed to high levels of oxidized low-density lipids (oxLDL) exhibit higher COX-2 (29). Thus, COX-2-inhibitors may be a promising approach because they inhibit synthesis of prostaglandin E_2_ (PGE_2_), which acts as a stimulator of indoleamine-2,3-dioxygenase [IDO; (30)] and mediates inflammatory response (31). IDO activation, in turn, promotes the conversion of tryptophan along the kynurenine pathway instead of serotonin and may explain the serotonin depletion and neurodegeneration hypotheses of depression (20, 32). Interestingly, depressed patients showed increased serum PGE_2_ (33).

Several reviews and meta-analyses have evaluated the efficacy of anti-inflammatory treatments in MDD patients, mostly concluding an overall limited beneficial effect for clinical outcome (34–39). These overview articles included four important trials that investigated the efficacy of celecoxib (a COX-2-inhibitor) augmentation to sertraline, fluoxetine, or reboxetine and showed a greater decline of symptom severity as compared to add-on placebo [although both groups showed a significant symptom reduction; (40–43)]. Given the notion that inflammatory activation is present only in a subgroup of patients, a critical point in former analyses is the evaluation of efficacy without addressing differential inflammatory levels and the evaluation of the relation between inflammatory compounds and response across treatment arms. Consequently, potential differentiating effects of inflammatory status by treatment were lost, which is reflected by the variability of, discrepancy between, or lack of positive individual study results (4, 44). Thus, it is necessary to investigate efficacy of different treatment regimens with respect to the levels of inflammatory compounds. A systematic review revealed that higher biomarker levels (IL-6, CRP, TNFα) were associated with treatment resistance to predominantly serotonergic acting drugs and that response improved using mainly noradrenergic or dopaminergic acting drugs, as well as using anti-inflammatory drugs (45). Further, when levels of these biomarkers were low, response to several anti-inflammatory agents was even lower as compared to placebo (45). Thus, studies need to compare levels of inflammatory biomarkers depending on response status per treatment arm is needed to gain more insight into patient profiles, which may help to individualize treatment options.

Here, we evaluated data from a trial designed to investigate the relation between levels of inflammatory compounds and response to add-on placebo vs. add-on celecoxib to standard selective serotonin reuptake inhibitor (SSRI) sertraline in MDD patients before reaching the state of treatment resistance (patients with no more than two unsuccessful treatments). In this report, we exploratively investigated serum MIF, neopterin, and TNFα levels because these compounds represent markers of macrophage and inflammatory activation. An earlier study by our group found elevated MIF levels in MDD patient, but it did not focus on patient subgroups (22). Other than that, to our knowledge MIF and neopterin have not yet been investigated in this context. Additionally, TNFα levels emerged as predictor of response to a TNF antagonist in depression (46), thus we were interested in studying this effect with celecoxib. Further, we also analyzed efficacy data independent from biomarker levels, as well as patient characteristics that are known to be related to inflammation and cardiovascular risk [i.e., smoking status, sex, body mass index (BMI), and age; (47–49)].

## Materials and Methods

This phase IIa study used a randomized, double-blind, placebo-controlled, parallel group design was used. Considering a high and variable placebo response in depression, a placebo control was chosen. Patients and investigators/study staff were blinded to treatment allocation. Psychiatric inpatients were sampled by convenience and randomly assigned in a 1:1 ratio by fixed block randomization to 6 weeks of either sertraline plus placebo or sertraline plus celecoxib treatment. The study was approved by the ethics committee of the medical faculty of Ludwig-Maximilians-University Munich (project-nr. 234-09). The trial was performed in compliance with the standards of good clinical practice and in accordance with the Declaration of Helsinki and its subsequent revisions. All participants provided written informed consent.

### Participants

Patients aged between 18 and 60 years were included if they had been diagnosed with MDD by a psychiatrist (DSM-IV-TR) and had a baseline Montgomery Åsberg Depression Rating Scale [MADRS; (50)] score of 20 or above (indicating moderate to severe depression). Exclusion criteria were as follows: comorbid psychotic depression, bipolar disorder, addiction, schizoaffective disorder, schizophrenia, and other psychiatric disorders if their symptomatology was predominating. Also excluded were patients taking concomitant psychotropic drugs or anti-inflammatory pain medication such as COX-2-inhibitors, non-steroidal anti-inflammatory drugs (NSAIDs), or paracetamol; pregnant or breastfeeding women; patients with history of cardiovascular disease or heart disease, or with current cardiovascular disturbances; and patients with inflammatory or other relevant diseases were excluded from the study. For the full inclusion and exclusion criteria see Supplementary Table A.

### Measures

Serum levels of MIF, neopterin, and TNFα were assessed at baseline and endpoint (week 6). Blood was drawn in fasting condition. All parameters were determined by enzyme-linked immunosorbent assay (ELISA) with standard curve by using the quantitative sandwich enzyme immunoassay technique (MIF, TNFα) and competitive enzyme immunoassay technique (neopterin), according to the instruction of the kit manufacturer (MIF ELH-MIF, RayBio®, Peachtree Corners, USA; TNFα HSTA00D, R&D Systems, Minneapolis, USA; neopterin EIA-2949, DRG International, Inc., Springfield, USA) and analyzed using MARS data analysis software (BMG Labtech, Ortenberg, Germany). BMI was calculated as body weight (in kg) divided by the square of body height (in m). Depression severity was assessed by trained raters at baseline and endpoint (6 weeks) with the MADRS (50, 51). Response was defined as a reduction of MADRS score of at least 50% on at endpoint, depending on the individual baseline score, and remission was defined as a score of 7 or lower at endpoint (52).

### Treatment Protocol

All patients who were taking antidepressant medication prior to the study underwent a 3-day wash-out period before the start of trial treatment. In case of premedication with long half-life, a longer period since the last treatment was necessary to be included (see Supplementary Table A). If needed, lorazepam was administered up to 4 mg/day during wash-out and for the first 2 weeks, up to 3 mg/day for the third week, up to 2.5 mg/day for the fourth and fifths weeks, and up to 1.5 mg/day for the sixth week. Zopiclone was administered up to 7.5 mg/nightly during the wash-out period (one patient had 15 mg) and the study time, if needed. At baseline, eligible patients were randomized to one of the following treatment arms: 50–100 mg sertraline daily (one tablet/unblinded) plus celecoxib twice daily (one capsule/200 mg/ blinded) or 50–100 mg sertraline daily (one tablet/unblinded) plus placebo twice daily (one capsule/blinded) The placebo capsule contained 308 mg microcrystalline cellulose coated by hard gelatin and was of the same size, weight, color, and shape as the celecoxib capsule. If a higher clinical benefit was expected, a dose of 150 mg sertraline was allowed.

### Statistics

SPSS software (IBM SPSS Statistics 25) was for statistical analyses used. The trial data presented here were planned as secondary per-protocol analyses. In this report, the primary outcomes were response and remission independently of inflammatory status and cytokine serum levels according to the response and remission status. Secondary analyses, investigated the role of BMI, age, sex, and smoking status. Endpoint parameter values were subtracted from baseline parameter values to reflect the change of values over time. Therefore, positive values represent a decrease and negative values represent an increase of levels over time. Descriptive analyses are given as mean and standard deviation or median and interquartile range for continuous variables, and as frequencies (percentages) for categorical variables. Testing for significances was performed using Chi-square test for categorical data (in case of 5 or fewer observations in the cells of the contingency table, Fisher's exact test was performed), Student's *t*-test for continuous data that met the criteria for parametric testing (in case of inhomogeneous variances, Welch-test is reported; in case of non-normality, Mann-Whitney-*U*-test was performed), or linear regression. To compare levels of inflammatory compounds between subgroups, Mann-Whitney-U-test for independent samples and Wilcoxon signed-rank test for dependent samples were used if data did not meet assumptions for parametric testing and/or small subsamples were tested. The significance level was set at 5% (two-sided) for all tests. As analyses were exploratory and subsamples were small, adjustment for multiple testing and correction for possible confounding variables were neglected in the primary analyses. Hence, the data reported here are preliminary. Due to statistical power limitations, trends are also reported (*p* < 0.10). The flow chart is given in Supplementary Figure 1.

## Results

### Patient Characteristics and Levels of Immune Parameters

Table 1 shows the descriptive demographic data, depression severity, and baseline biomarker levels in each treatment group. Table 2 shows the levels of immune parameters at baseline and endpoint in the placebo- and celecoxib-treated groups, as well as for responders/remitters and non-responders/non-remitters. Because drug treated patients were randomized but sample is of limited size, demographic data and baseline parameter values were tested for differences between the treatment arms. No statistically significant differences emerged for MADRS baseline score, MADRS endpoint score, percentage MADRS score reduction over time, age, baseline BMI, BMI at endpoint, sex distribution, smoking status, and immune parameter values at baseline between placebo and celecoxib groups (see Supplementary Table B).

**Table 1:** Descriptive characteristics of patients with MDD.

	**Sertraline** **+** **placebo**		**Sertraline** **+** **celecoxib**	
MADRS baseline *Md (IQR)*	29.00 (4.00)	*N* = 23	28.00 (8.00)	*N* = 19
MADRS endpoint *M (SD)*	12.35 (7.75)	*N* = 23	13.20 (7.21)	*N* = 20
% MADRS score reduction *M (SD)*	58.91 (23.05)	*N* = 23	52.16 (26.25)	*N* = 19
Age *M (SD)*	38.78 (10.71)	*N* = 23	39.25 (12.75)	*N* = 20
BMI baseline *M (SD)*	23.28 (3.46)	*N* = 23	23.31 (3.22)	*N* = 20
BMI endpoint *M (SD)*	22.58 (3.26)	*N* = 21	22.82 (2.93)	*N* = 19
Sex women *N* (%)	12/23 (52.17)		9/20 (45.00)	
Smoking yes *N* (%)	7/23 (30.43)		11/20 (55.00)	
MIF (pg/ml) *Md (IQR)*	3484.00 (5002.25)	*N* = 22	4306.00 (4638.25)	*N* = 18
Neopterin (ng/ml) *Md (IQR)*	0.85 (0.47)	*N* = 22	0.73 (0.49)	*N* = 19
TNFα (pg/ml) *Md (IQR)*	0.78 (0.81)	*N* = 19	0.76 (0.71)	*N* = 15

*BMI, body mass index; MADRS, Montgomery Åsberg Depression Rating Scale; Md, median; IQR, interquartile range; M, mean; SD, standard deviation. MADRS score at baseline was available in only 19 of the 20 patients in the celecoxib group but was available in all 20 patients at endpoint assessment*.

**Table 2:** Baseline and endpoint biomarker levels according to treatment and response status.

	**Baseline *Md* (*IQR*)**	**Week 6 *Md* (*IQR*)**	***Z* (*N*)**	***p***
**MIF (pg/ml)**				
Placebo	3484.00 (5002.25)	2102.00 (2862.50)	−1.61 (22)	0.11
Celecoxib	3406.00 (4638.25)	4197.50 (3826.75)	−0.28 (18)	0.78
Responder	3484.00 (4948.25)	3078.00 (4820.00)	−0.60 (26)	0.55
Non-responder	4092.00 (5301.50)	3519.00 (2888.00)	−0.73 (13)	0.46
Remitter	2663.00 (3.697)	1207.50 (2893.00)	−1.96 (12)	0.05^+^
Non-remitter	4321.50 (5545.75)	3736.50 (4160.50)	−0.18 (28)	0.86
**Neopterin (ng/ml)**				
Placebo	0.85 (0.47)	0.79 (0.44)	−0.52 (22)	0.60
Celecoxib	0.73 (0.49)	0.88 (0.21)	−2.15 (19)	0.03*
Responder	0.72 (0.50)	0.86 (0.38)	−1.21 (26)	0.23
Non-responder	0.87 (0.38)	0.91 (0.36)	−1.67 (14)	0.10
Remitter	0.80 (0.48)	0.81 (0.47)	−0.08 (12)	0.94
Non-remitter	0.76 (0.51)	0.88 (0.36)	−2.12 (29)	0.03*
**TNFα** **(pg/ml)**				
Placebo	0.78 (0.81)	0.92 (0.89)	−1.25 (19)	0.21
Celecoxib	0.76 (0.71)	0.78 (1.02)	−0.34 (15)	0.73
Responder	0.71 (0.85)	0.68 (0.94)	−0.57 (21)	0.57
Non-responder	0.96 (0.96)	1.08 (0.96)	−0.11 (13)	0.92
Remitter	0.75 (0.90)	0.63 (0.59)	−0.15 (10)	0.88
Non-remitter	0.81 (0.86)	1.07 (1.00)	−0.69 (24)	0.49

Md, median; IQR, interquartile range; Z-test statistic Wilcoxon signed rank test. *p < 0.05 and ^+^p < 0.10.

### Response and Remission Rates

Table 3 shows the proportions of responders and remitters in each treatment arm. No significant differences emerged for the distribution of response rates (χ^2^ = 0.62; *p* = 0.43) or remission rates (χ^2^ = 0.49; *p* = 0.49) between the two treatment groups. Both treatment groups showed a significant decline of MADRS scores over time (placebo: *T* = 12.81; *SE* = 1.31; *p* < 0.001; 95% *CI* = [14.07; 19.51]; celecoxib: *T* = 7.86; *SE* = 2.00; *p* < 0.001; 95% *CI* = [11.53; 19.94]). In responders, the MADRS score decreased slightly more in the celecoxib group than in the placebo group, although the difference was not significant (*T* = −1.15; *SE* = 1.75; *p* = 0.26; 95% *CI* = [−5.61; 1.60]). In non-responders, the MADRS score decreased slightly more in the placebo group than in the celecoxib group, but this difference was also not significant (*T* = 1.01; *SE* = 2.45; *p* = 0.33; 95% *CI* = [−2.81;7.77]).

**Table 3:** Response and remission rates of MDD patients in each treatment arm.

	**Responder**	**Non-responder**
Sertraline + placebo % (*N*)	69.6 (16)	30.4 (7)
Sertraline + celecoxib % (*N*)	57.9 (11)	42.1 (8)
	**Remitter**	**Non-remitter**
Sertraline + placebo % (*N*)	34.8 (8)	65.2 (15)
Sertraline + celecoxib % (*N*)	25.0 (5)	75.0 (15)

### Predictive Capability of Biomarkers for Response and Remission

#### MIF

**Figure 1:**
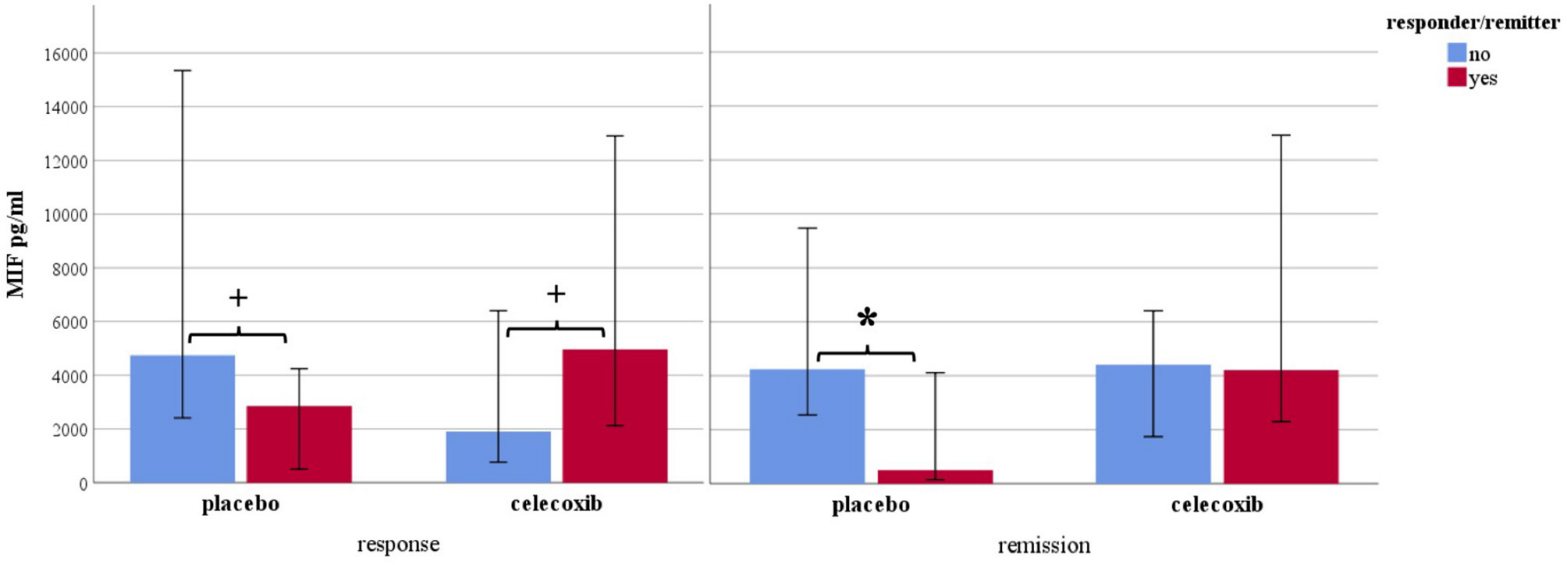
Macrophage migration inhibitory factor levels at baseline according to response and remission status. Median levels are displayed in pg/ml; 95% confidence interval error bars are displayed; *placebo* sertraline plus placebo group; *celecoxib* sertraline plus celecoxib group. *p < 0.05 and ^+^*p* < 0.10.

Figure 1 shows the results for MIF at baseline. In the placebo group (sertraline only), responders showed a trend for lower MIF levels at baseline compared with non-responders, and remitters showed significantly lower MIF levels at baseline than non-remitters. In the celecoxib group (sertraline plus celecoxib), responders showed a trend for higher MIF levels at baseline compared with non-responders, but no significant difference emerged between remitters and non-remitters. Statistical test results are shown in Table 4A. At endpoint, MIF levels were not significantly different between responders and non-responders in the placebo group but were significantly lower in remitters compared with non-remitters. In the celecoxib group, responders did not differ statistically from non-responders at endpoint, but remitters showed a trend for lower MIF levels than non-remitters. Statistical test results are shown in Table 4B.

**Table 4A:** Comparison of baseline MIF levels of different response statuses in the two treatment arms.

Sertraline +	Responder (*Md*)	Non-responder (*Md*)	*U*	*p*
placebo	2853.00 pg/ml	4743.00 pg/ml	29.00	<0.10^+^
	Remitter (*Md*)	Non-remitter (Md)	*U*	*p*
	501.00 pg/ml	4240.00 pg/ml	15.00	<0.01**
Sertraline +	Responder (*Md*)	Non-responder (*Md*)	*U*	*p*
celecoxib	4961.00 pg/ml	1901.00 pg/ml	15.00	0.07^+^
	Remitter (*Md*)	Non-remitter (*Md*)	*U*	*p*
	4209.00 pg/ml	4403.00 pg/ml	24.00	0.40

Md, median; U-Mann-Whitney-U-test statistic. **p < 0.01 and ^+^p < 0.10.

**Table 4B:** Comparison of endpoint MIF levels of different response statuses in the two treatment arms.

Sertraline +	Responder (*Md*)	Non-responder (*Md*)	*U*	*p*
placebo	1066.00 pg/ml	2924.00 pg/ml	37.00	0.28
	Remitter (*Md*)	Non-remitter (Md)	*U*	*p*
	480.00 pg/ml	2924.00 pg/ml	8.00	<0.01**
Sertraline +	Responder (*Md*)	Non-responder (*Md*)	*U*	*p*
celecoxib	3970.00 pg/ml	4814.00 pg/ml	32.00	0.92
	Remitter (*Md*)	Non-remitter (*Md*)	*U*	*p*
	3579.00 pg/ml	4587.00 pg/ml	15.00	0.09^+^

Md, median; U-Mann-Whitney-U-test statistic. **p < 0.01 and ^+^p < 0.10.

Regarding the change of MIF levels over time, in the placebo group non-responders showed a trend for a decrease to endpoint (*Z* = −1.86; *p* = 0.06), but no differences of MIF change between placebo responders and non-responders were found. However, the celecoxib group showed a trend for differential change of MIF levels over time: Remitters showed a decrease while non-remitters showed an increase (*U* = 14.00; *p* = 0.07). Furthermore, the change in MIF levels was significantly different in non-responders and non-remitters to celecoxib (increase) compared with non-responders and non-remitters to placebo (decrease; *U* = 6.00; *p* = 0.03 and *U* = 51.00; *p* = 0.03, respectively).

#### Neopterin

No significant differences in neopterin levels emerged at baseline and endpoint between responders and non-responders as well as remitters and non-remitters in either treatment arm (see Supplementary Tables C.1, C.2). Investigating change over time, only non-responders to celecoxib showed a trend for an increase of neopterin levels (*Z* = −1.95; *p* = 0.05).

#### TNFα

No significances emerged for TNFα levels at baseline. At endpoint, only lower levels were found in placebo responders compared with placebo non-responders. For further details, see Supplementary Tables D.1, D.2. Investigating change over time, placebo non-responders showed a trend for an increase of TNFα levels over time (*Z* = −1.86; *p* = 0.06). In contrast, celecoxib non-responders showed a decrease of TNFα levels over time (*Z* = −2.21; *p* = 0.03). In the celecoxib group, responders showed differential course over time (increase) compared with non-responders (decrease; *U* = 8.00; *p* = 0.03). In non-responders, celecoxib-treated patients showed a decrease of TNF α levels over time whereas placebo-treated patients showed an increase (*U* = 1.00; *p* < 0.01).

### Patient Characteristics Associated With Inflammation

The most interesting and relevant results were obtained for MIF. Therefore, possible confounders were further investigated.

#### BMI

No significant correlation was found between BMI and MIF baseline levels (Spearman-*Rho* = 0.23; *p* = 0.16). We found no difference in the change of BMI over time according to treatment (*T* = −0.54; *p* = 0.59) or response/ remission status across treatments (*T* = −0.44; *p* = 0.66 and *T* = 0.15; *p* = 0.88, respectively) occurred. However, in the placebo group a decrease in BMI over time significantly predicted a lower response (% MADRS score reduction) [*R*^2^ = 0.29; *F*_(1, 19)_ = 7.85; ß = −22.37; *p* = 0.01].

#### Age

We found a significant positive correlation between age and MIF levels (Spearman-*Rho* = 0.34, *p* = 0.03) at baseline. No difference emerged in age according to response/ remission status (*T* = −0.56; *p* = 0.58 and *T* = −1.04; *p* = 0.31, respectively) across treatments. However, higher age significantly predicted a lower response (% MADRS score reduction) in the placebo group [*R*^2^ = 0.19; *F*_(1, 21)_ = 4.76; ß = −0.93; *p* = 0.04].

#### Sex

Men had significantly higher MIF levels (*U* = 115.00; *p* = 0.02) at baseline than women. Response/remission status were independent from sex (response: χ^2^ = 0.10; *p* = 0.75; remission: χ^2^ = 0.19; *p* = 0.67). Response status was also statistically independent from sex in the placebo and celecoxib subgroups (*p* > 0.99 and *p* = 0.65, respectively). However, in the celecoxib group men showed a numerically lower reduction in MADRS score than women. Regarding changes in biomarker level over time, women as compared to men showed a significantly different course of MIF levels (women: increase, men: decrease; *U* = 122.00; *p* = 0.04). This difference emerged only in the placebo group (*U* = 25.00; *p* = 0.02) and was seen in responders as a trend (*U* = 47.00; *p* = 0.06).

#### Smoking Status

No significant differences in baseline MIF levels emerged between smokers and non-smokers (*U* = 164.00; *p* = 0.44). Furthermore, response/remission status were independent from smoking status (response: χ^2^ = 0.14; *p* = 0.71; remission: χ^2^ = 0.14; *p* = 0.71). Response status was also independent from smoking status in the placebo and celecoxib subgroups (*p* = 0.63 and *p* = 0.66, respectively).

#### Adjusted Analysis

Because MIF levels were associated with age and sex, we performed preliminary regression analyses with age and sex as covariates to further investigate the predictive capability of response status on the expression of MIF levels. In the placebo group, a trend emerged for predicting of MIF levels at baseline by response status, age, and sex [*R*^2^ = 0.32; *F*_(3, 18)_ = 2.82; *p* = 0.07]. For remission, a significant model was obtained [*R*^2^ = 0.37; *F*_(3, 18)_ = 3.56; *p* = 0.04]. Similarly, the model predicting MIF levels at endpoint was significant [*R*^2^ = 0.41; *F*_(3, 18)_ = 4.19; *p* = 0.02]. In the celecoxib group, a trend emerged for the prediction of MIF levels at baseline by response status, age, and sex [*R*^2^ = 0.38; *F*_(3, 13)_ = 2.60; *p* < 0.10]. Data on the individual predictors are given in Supplementary Table D. The results of the adjusted analyses are mostly in accord with the data given under 3.3. Since some models revealed a significant *R*^2^ but partly lacking significance of single predictors, collinearity diagnostics were performed. No indication for multicollinearity was present in any of the models. Thus, this effect may result from the collective impact of more or less nearly significant predictors.

## Discussion

Overall, no difference in response or remission rates between the two treatment arms were found. One reason for this result might be that celecoxib add-on to sertraline is not superior to sertraline plus placebo. In contrast to our findings, a meta-analysis of previous trials found a superior effect of add-on celecoxib to standard antidepressant treatment over add-on placebo without taking inflammatory status into account (53, 54). Noteworthy, the magnitude of symptom reduction and the obtained response rates varied among the studies, even despite using the same celecoxib dose (41, 53). The studies with very high response rates to celecoxib had a higher women to men ratio in the celecoxib group than the other studies and our study (41, 42, 53). According to previous literature, inflammatory activation is associated with depression severity particularly in women (55), thus leading to a higher potential of benefitting from anti-inflammatory therapy. In line with our study, another trial investigating add-on celecoxib to SSRI compared to placebo plus SSRI in drug-naïve depressed women and found no difference in reduction of depression severity at endpoint (41). However, the authors found a superior effect of celecoxib after half the treatment phase, i.e., 4 weeks, suggesting that celecoxib might accelerate symptom reduction during early treatment phases (41). Although celecoxib add-on was not superior to standard treatment in our study, both groups had a reasonably large proportion of responders and both treatment groups showed a significant decline of depression severity over time. As compared to one of the investigated trials in the meta-analysis, we found a similar response rate in the celecoxib group while both studies used sertraline and the same celecoxib dose (43). However, the non-superiority of add-on celecoxib in our trial questions the clinical benefit that the previous studies concluded. Because only a subgroup of patients exhibits an increased pro-inflammatory profile and patients were not stratified for inflammatory state before receiving anti-inflammatory treatment, it may be expected to find no substantial difference in response rates. This assumption is supported by studies on other (add-on) anti-inflammatory treatments, which showed that depressed patients with a low inflammatory status exhibited even lower response rates after anti-inflammatory therapy than patients with a low inflammatory status who had received (standard therapy plus) placebo (45). Therefore, patients with low levels of inflammation may have contributed to lower response rates to celecoxib. Furthermore, our study found much higher response and remission rates in the sertraline group than were found in the control groups in most of the other trials (53). In our study, almost all patients were without current premedication and about half the patients were experiencing their first episode of depression. Sertraline is a potent antidepressant and drug-naïve patients respond better to treatment than patients who have received multiple treatments (2), which together may explain the high response and remission rates to sertraline plus placebo in our study. Furthermore, many of the above-mentioned studies included outpatients, whereas our sample consisted only of inpatients (41–43). A high placebo effect may arise from receiving extensive care and attention in a hospitalized setting. Generally, given the variability of previous study results, explanations for differences of our results are rather speculative. Regarding change of biomarker levels over time, significant results for non-responders and responders or non-remitters and remitters across treatment arms, and for sertraline with add-on placebo or add-on celecoxib across response status are lacking not indicating any general effects of treatment or response status for change of biomarker levels.

Our study goes beyond the current state of knowledge by looking into response status in subgroups. A dependency of inflammatory biomarker levels and response/remission to different treatment becomes apparent, at baseline and during the course of treatment. However, a clear pattern was only observed for MIF, which is consistent with previous literature. Non-responders to sertraline plus placebo showed a trend for higher baseline levels as compared with sertraline responders. This was especially and significantly evident in non-remitters compared with remitters pointing to the well-known treatment resistance to standard serotonergic agents when pro-inflammatory levels are increased (45). Vice versa, responders to celecoxib showed a trend for higher baseline MIF levels as compared with celecoxib non-responders (who had the overall lowest levels), suggesting a beneficial effect of anti-inflammatory medication on clinical outcome when such activation is present. Interestingly, baseline MIF levels in non-responders/non-remitters to sertraline plus placebo were similar to those of responders/remitters to sertraline plus celecoxib. This oppositional relationship has also been found for CRP, IL-6, and IL-1ra (46, 56, 57). Moreover, higher MIF baseline mRNA levels were also shown to predict response to escitalopram or nortriptyline in depressed patients (58). The difference between baseline levels in placebo remitters vs. non-remitters was larger than between responders/non-responders and was still present at the end of the study. We therefore conclude that stronger or faster symptom reduction can be achieved with standard SSRIs when MIF levels are preferably low and high MIF levels after treatment indicate the persistence of clinically relevant depressive symptoms. Celecoxib non-responders and non-remitters showed the highest MIF levels of all subgroups at endpoint (see Table 4B). This change was significantly different from that in non-responders and non-remitters in the placebo groups and may point to a subgroup of concern that should be studied in more detail in the future. Taken together, MIF shows a predictive capability for remission (and a trend toward such capability for response) in treatment as usual (SSRI) and a trend toward predicting response to add-on celecoxib.

Regarding relevant patient characteristics, MIF levels were found to be related to age and sex but not to BMI. In the placebo group, weight loss and higher age were associated with treatment resistance. Since age was positively associated with both, MIF levels and treatment failure, the concept of immunosenscence presents a suitable explanation. Immunosenscence describes the changes of the immune system which, among others, is characterized by low-grade inflammation and all of which increase during aging (59). It is thus not surprising that these factors were related in our study. We found no effect of sex on response and no associations with smoking whatsoever. Hence, BMI, sex, and age seem to be linked to MIF levels and/or response status and should be addressed in design and statistical evaluation of future studies.

As for neopterin, baseline levels did not discriminate between responders/remitters and non-responders/non-remitters to either treatment. Previous research found that higher neopterin levels were associated with depression and the number of depressive episodes (21, 60) indicating higher disease severity. This also demonstrates that neopterin levels seem to rise with treatment resistance as the increase in non-remitters shows in our study. Further, this may be especially driven by the celecoxib non-remitters explaining the increase of neopterin levels in the whole celecoxib group (see Table 2 and Supplementary Tables C.1, C.2). To our knowledge, no other studies have investigated baseline neopterin in relation to treatment response yet. Our study found no indication that neopterin is a potential biomarker. However, this result should be verified in a study with a larger sample size and greater power. TNFα levels were not significantly different at baseline but at endpoint in the placebo group indicating that treatment resistance to standard SSRI is accompanied by high levels of TNFα. One earlier study found significantly lower TNFα levels at baseline in responders to sertraline (at least 50% MADRS score reduction) compared with non-responders (61). Our data showed the same tendency, but the difference did not reach statistical significance. The authors of the earlier study (61) defined endpoint at 12 weeks which might better separate response status and biomarker levels. Further, Powell et al. (62) found higher levels of TNF gene expression in SSRI (escitalopram) non-responders compared to responders and this difference was even larger at endpoint (8 weeks) than at baseline, supporting our results. However, conflicting results exist (63, 64).

The involvement of the kynurenine pathway in MDD pathophysiology has been receiving growing attention. In fact, pro-inflammatory cytokines such as IFNγ, TNFα, IL-1, and the hormone PGE2, which is stimulated by MIF via COX-2-upregulation, stimulate the activity of the enzyme IDO and consequently the breakdown of tryptophan into potentially neurotoxic kynurenine metabolites (13, 65–68). This results in a lack of serotonin, a widely known characteristic of MDD. Because MIF stimulates PGE2 production, which is counteracted by a celecoxib-related decrease in IDO synthesis, MIF might act as a surrogate for COX-2 and PGE2 activity. Further, TNFα activates IDO (13), also possibly explaining the lacking serotonergic response. Furthermore, kynurenine in turn may lead to more pro-inflammatory cytokine release like TNFα (69) possibly explaining the higher levels of TNFα at endpoint. In fact, in all three markers we observed the same numeric trend of higher levels in non-responders/non-remitters at baseline and at endpoint, though not all the differences reached statistical significance. Nevertheless, this points in the direction of disturbed antidepressant action in those patients. Other previous results show that higher kynurenine/tryptophan ratio (favors kynurenine pathway) was predictive of remission after celecoxib add-on treatment in another sample of MDD patients (70). With perspective on the comorbidity of depression and cardiovascular disease, IDO activity is associated with cardiovascular risk factors (71), and celecoxib has shown beneficial effect on atherosclerotic progression (72).

This study has several limitations. Analyses in small subsamples have limited statistical power and non-significant results may therefore be a matter of type II error, which is we refrained from confident interpretation. However, we did give statistical trends some credibility. There is an ongoing debate on whether the *p*-value should be treated as a strict cut-off, and many scientists favor a non-categorical use today (73). The addition of covariates to a binary predictor in the adjusted analyses further limits power while the smaller sample size already increases variance. Future studies should aim at replicating these findings in larger samples which would also allow for multiple test correction. Due to the preliminary nature of subgroup analyses, and especially the adjusted analyses, results are rather hypothesis generating for future studies. We used convenience sampling so that even though important patient characteristics were equally distributed between the treatment arms other variables might have acted as confounders that were not accounted for. For example, childhood adverse experience was shown to be related to cytokine levels in depression (74). In addition, our sample consisted of inpatients only, so the results cannot be generalized to outpatient settings. Because antidepressant medication has some immunomodulatory effects (75), prior use of such medication might have elicited a modulating effect before the study already. Further, we did not evaluate kynurenine metabolites which will be important for future analyses to demonstrate mechanistic links as discussed above. In general, one important drawback is that there are no established cut-offs yet for categorizing levels of the investigated biomarkers as high or low. Such cut-off values are needed so that patients can be stratified by inflammatory level beforehand to investigate response to a tailored treatment allocation.

## Conclusion

Celecoxib add-on did not lead to greater response rate at 50% symptom reduction than sertraline plus placebo regardless of inflammatory state, but patients were not stratified beforehand according to their level of pro-inflammatory activation. MIF shows potential for acting as a reliable biomarker indicating treatment responsiveness, especially remission. Response rates may be increased if such biomarkers were used to guide treatment choice or change when their monitoring during treatment indicates non-response. The present study serves as a call for future investigations, in particular on treatment remission in response to anti-inflammatory vs. standard antidepressant treatments, stratifying patients by immune activation in advance. As the trends found for response status cannot be neglected entirely, studies should also reevaluate these finding in larger samples. Therefore, cut-off values should also be established for classifying abnormal immune activation. Moreover, complex models including possible confounding variables should be performed and more biomarkers should be investigated in larger samples to predict response.

## Data Availability Statement

The raw data supporting the conclusions of this article will be made available by the authors upon request, without undue reservation.

## Ethics Statement

The studies involving human participants were reviewed and approved by Ethical Committee of the Medical Faculty of Ludwig-Maximilians-University Munich, Germany (project-nr. 234-09). The patients/participants provided their written informed consent to participate in this study.

## Author Contributions

MS has prepared the data and conducted the analysis, evaluation, and interpretation of the present work, as well has drafted the present manuscript. BB and GA-H have conducted data acquisition. EW has contributed to planning the study and conducted data acquistion. PZ has conducted the laboratory analyses. RM has contributed to the interpretation of data. HD has contributed to design and planning the study and data interpretation. NM has contributed to design and planning the study, data acquisition, and evaluation. BB, EW, GA-H, PZ, RM, HD, and NM have critically reviewed and revized the content. All authors contributed to the manuscript and approved submission.

## Funding

This present work was funded by the EU 7th Framework program (grant number EU-FP7-CP-IP-2008-222963/EU-FP7-PEOPLE-2009-IAPP-MarieCurie-286334) and Horizon 2020 (grant number H2020-SC1-2016-2017/H2020-SC1-2017-Two-Stage-RTD). Further, part of this work was supported by the foundation Immunität und Seele. The funders had no role in the study design, data collection and analysis, or decision to publish.

## Conflict of Interest

RM declares personal fees from Otsuka/Lundbeck outside the submitted work. HD is the coordinator of the project funded by the EU. The remaining authors declare that the research was conducted in the absence of any commercial or financial relationships that could be construed as a potential conflict of interest.

## Publisher's Note

All claims expressed in this article are solely those of the authors and do not necessarily represent those of their affiliated organizations, or those of the publisher, the editors and the reviewers. Any product that may be evaluated in this article, or claim that may be made by its manufacturer, is not guaranteed or endorsed by the publisher.
