# Supplementary material for: Efficacy of Sertraline Plus Placebo or Add-On Celecoxib in Major Depressive Disorder: Macrophage Migration Inhibitory Factor as a Promising Biomarker for Remission After Sertraline—Results From a Randomized Controlled Clinical Trial
Source: Front Psychiatry. 2021 Sep 27;12:615261. doi: 10.3389/fpsyt.2021.615261 (PMC8504576; doi:10.3389/fpsyt.2021.615261)
Supplement: Supplementary file 1 [file Data_Sheet_1.pdf]

Supplementary Material

1 Supplementary Figure and Tables

1.1 Supplementary Figure

Supplementary Figure 1. Patients flow-chart according to CONSORT guideline.

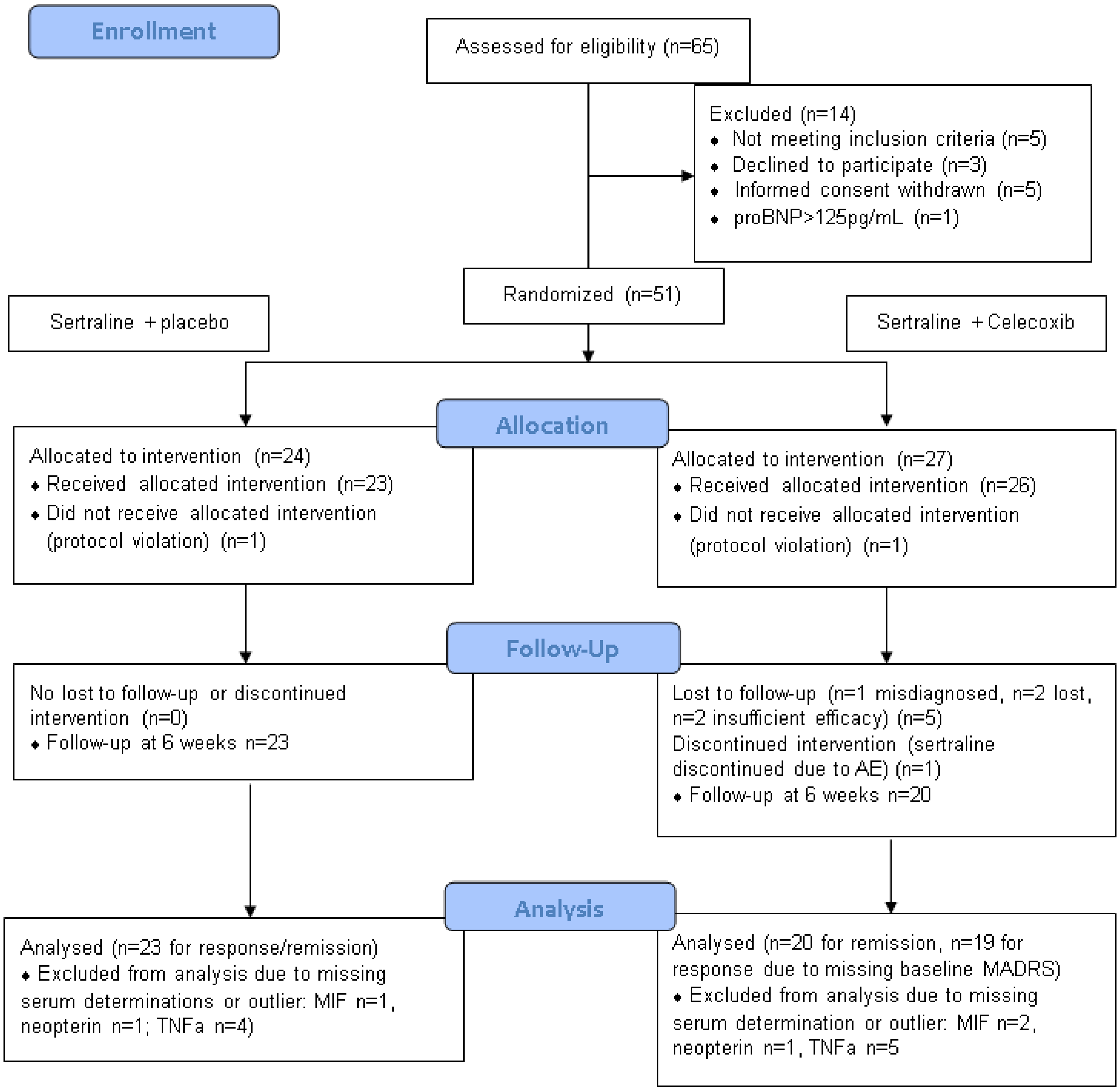

1.2 Supplementary Tables

Supplementary Table A. Inclusion and exclusion criteria of the patient population.

	Inclusion criteria
(1)	Major depression diagnosed by psychiatrist.
(2)	DSM IV TR: 296.2x single depressive episode or 296.3x recurrent depressive episode.
(3)	For the present evaluation MADRS score ≥ 20 was used representing moderate to severe depression severity. Originally HamD-17 score ≥ 22 was defined; no subject was excluded due to exchange of rating scale criterion.
(4)	Informed consent.
(5)	Age between 18 and 60 years.
	Exclusion criteria
(1)	Psychotic depression or bipolar disorder, drug or alcohol addiction, schizoaffective disorders, schizophrenia. Other disorders (e.g. obsessive compulsive disorder, anxiety disorder, personality disorder) in case the symptoms predominate the clinical picture.
(2)	Unsuccessful treatment with more than 2 antidepressant medications (at therapeutically adequate doses and duration) during current episode.
(3)	Concomitant use of psychotropic drugs, including mood stabilizers, besides defined co-medication.
(4)	Immediate risk for suicidal behavior (3 on HamD-17 rating scale or 5 on MAD Rating Scale).
(5)	Women who are pregnant, breast feeding or planning to become pregnant during the course of study.
(6)	Women who are not post-menopausal (no natural menopause established in retrospect after 12 consecutive months of amenorrhea without hormone replacement therapy during the last 5 months), surgically sterilized or using a highly effective method of contraception (an implanted or injected hormonal contraceptive, some intrauterine contraceptive devices (IUDs) containing hormones, sexual abstinence, or have a vasectomized partner). Females using combined oral contraceptives should use a different or additional highly effective method of contraception as listed above.
(7)	Any history of cardiovascular disease (e.g. angina, heart attack, stroke, congestive heart failure), uncontrolled high blood pressure, documented peripheral arterial insufficiency and symptomatic, clinically significant claudication, a history of peripheral arterial embolism or cerebrovascular disease.
(8)	Patients at risk of QT/QTc interval prolongation (QTc> 450 ms, family history of long QT syndrome or use of medication prolonging QT/QTc interval).
(9)	History of coronary heart disease (CHD) or any other heart disease.
(10)	Serum NTproBNP ≥ 125 pg/mL indicating (sub-) clinical heart failure.
(11)	History of upper or lower gastrointestinal (GI) ulceration, perforation and/or obstruction.
(12)	History of upper or lower GI bleeding within the previous year.
(13)	History of inflammatory bowel disease.
(14)	Undergoing cancer chemotherapy.
(15)	Known HIV infection or clinically manifest Acquired Immune Deficiency Syndrome (AIDS), diabetes, asthma, COPD, Parkinson's or Alzheimer's disease, or any other serious condition likely to interfere with the conduct of the trial.
(16)	Clinically relevant hepatic or renal impairment (serum albumin < 25 g/L or Child-Pugh > 10 or renal GFR < 30 mL/min), or other clinically significant physical findings or clinically significant laboratory results at screening or baseline, as determined by the investigator.
(17)	History of allergy to sertraline, celecoxib, sulfonamides or closely related compounds, or excipients.
(18)	History of hypersensitivity or intolerance to pain medications.
(19)	Use of pain medication, such as a COX-2 inhibitor, NSAID (non-steroidal anti-inflammatory drug, including aspirin) or acetaminophen (syn. paracetamol) within 72 hours prior to study entry (24 hours for short-acting drugs such as aspirin or acetaminophen).
(20)	Patients currently taking warfarin.
(21)	Participation in a study of an investigational drug or device concomitantly or within 30 days prior to this study.
(22)	Patients thought to be unreliable or incapable of complying with the requirements of the protocol.
(23)	Treatment with monoamino oxidase inhibitors during the last 14 days or treatment with fluoxetine during the last 6 weeks.

Supplementary Table B. Comparison of placebo and celecoxib treatment groups.

	test (N)	SE	p	CI
MADRS baseline	U=198.50 (42)		0.61	
MADRS endpoint	T=-0.37 (43)	2.29	0.71	[-5.49;3.78]
MADRS score reduction (%)	T=0.89 (42)	7.61	0.38	[-8.62;22.13]
age	T=-0.13 (43)	3.58	0.89	[-7.69;6.76]
BMI baseline	T=-0.03 (43)	1.02	0.97	[-2.10;2.04]
BMI endpoint	T=-0.25 (40)	0.99	0.80	[-2.24;1.75]
sex	χ^2=0.22 (43)		0.63	
smoking status	χ^2=2.65 (43)		0.10	
MIF (pg/ml)	U=175.00 (40)		0.53	
Neopterin (ng/ml)	U=174.50 (41)		0.36	
TNFα (pg/ml)	U=111.00 (34)		0.27	

Notes. *MADRS* Montgomery Åsberg Depression Rating Scale *SE* standard error *CI* confidence interval.

Supplementary Table C.1. Comparison of baseline neopterin levels of different response statuses in the two treatment arms.

sertraline + placebo	responder (*Md*)	non-responder (*Md*)	*U*	*p*
	0.80 ng/ml	0.90 ng/ml	38.00	0.31
	remitter (*Md*)	non-remitter (Md)	*U*	*p*
	0.55 ng/ml	0.90 ng/ml	29.00	0.10
sertraline + celecoxib	responder (*Md*)	non-responder (*Md*)	*U*	*p*
	0.61 ng/ml	0.73 ng/ml	34.00	0.68
	remitter (*Md*)	non-remitter (*Md*)	*U*	*p*
	0.99 ng/ml	0.59 ng/ml	23.50	0.29

Notes. *Md* median; *U* Mann-Whitney-U-test statistic.

Supplementary Table C.2. Comparison of endpoint neopterin levels of different response statuses in the two treatment arms.

sertraline + placebo	responder (*Md*)	non-responder (*Md*)	*U*	*p*
	0.66 ng/ml	0.94 ng/ml	33.50	0.18
	remitter (*Md*)	non-remitter (Md)	*U*	*p*
	0.66 ng/ml	0.90 ng/ml	33.00	0.17
sertraline + celecoxib	responder (*Md*)	non-responder (*Md*)	*U*	*p*
	0.94 ng/ml	0.88 ng/ml	35.00	0.75
	remitter (*Md*)	non-remitter (*Md*)	*U*	*p*
	0.95 ng/ml	0.86 ng/ml	27.50	0.49

Notes. *Md* median; *U* Mann-Whitney-U-test statistic.

Supplementary Table D.1. Comparison of baseline TNFα levels of different response statuses in the two treatment arms.

sertraline + placebo	responder (*Md*)	non-responder (*Md*)	*U*	*p*
	0.56 pg/ml	0.86 pg/ml	27.00	0.21
	remitter (*Md*)	non-remitter (Md)	*U*	*p*
	0.56 pg/ml	0.86 pg/ml	26.00	0.25
sertraline + celecoxib	responder (*Md*)	non-responder (*Md*)	*U*	*p*
	0.71 pg/ml	1.08 pg/ml	16.00	0.20
	remitter (*Md*)	non-remitter (*Md*)	*U*	*p*
	0.93 pg/ml	0.76 pg/ml	20.00	0.79

Notes. *Md* median; *U* Mann-Whitney-U-test statistic.

Supplementary Table D.2. Comparison of endpoint TNFα levels of different response statuses in the two treatment arms.

sertraline + placebo	responder (*Md*)	non-responder (*Md*)	*U*	*p*
	0.52 pg/ml	1.24 pg/ml	16.00	0.03*
	remitter (*Md*)	non-remitter (Md)	*U*	*p*
	0.52 pg/ml	1.05 pg/ml	27.00	0.29
sertraline + celecoxib	responder (*Md*)	non-responder (*Md*)	*U*	*p*
	0.83 pg/ml	0.60 pg/ml	17.00	0.24
	remitter (*Md*)	non-remitter (*Md*)	*U*	*p*
	0.73 pg/ml	1.10 pg/ml	18.00	0.60

Notes. *Md* median; *U* Mann-Whitney-U-test statistic; *p<0.05.

Supplementary Table E. Single predictor estimates of the preliminary adjusted analyses.

baseline MIF levels	*β*	*t*	*df*	*p*	*CI*
sertraline+placebo					
responder	-845.68	-0.35	18	0.73	[-5932.17;4240.82]
age in years	210.27	1.99	18	0.06⁺	[-12.04;432.57]
sex	-3479.53	-1.69	18	0.11	[-7810.65;851.59]
sertraline+placebo					
remitter	-3141.23	-1.28	18	0.22	[-8283.26;2000.80]
age	156.60	1.46	18	0.16	[-68.14;381.34]
sex	-3352.00	-1.69	18	0.11	[-7511.15;807.15]
sertraline+celecoxib					
responder	5223.36	2.14	13	0.05⁺	[-54.61;10501.33]
age	-49.13	-0.54	13	0.60	[-247.39;149.13]
sex	-4856.75	-2.19	13	0.047*	[-9644.32;-69.18]

endpoint MIF levels	*β*	*t*	*df*	*p*	*CI*
sertraline+placebo					
remitter	-3787.09	-3.51	18	0.003**	[-6057.32;-1516.86]
age	-91.05	-1.93	18	0.07⁺	[-190.27;8.17]
sex	562.60	0.64	18	0.53	[-1273.69;2398.88]

Notes. age in years; *β* regression coefficient; t test; df degrees of freedom; CI 95% confidence interval; ⁺p<0.10 *p<0.05 **p<0.01
